# Background Registration-Based Adaptive Noise Filtering of LWIR/MWIR Imaging Sensors for UAV Applications

**DOI:** 10.3390/s18010060

**Published:** 2017-12-27

**Authors:** Byeong Hak Kim, Min Young Kim, You Seong Chae

**Affiliations:** 1 School of Electronics Engineering, Kyungpook National University, 80 Daehakro, Bukgu, Daegu 41566, Korea; byeonghak81.kim@hanwha.com (B.H.K.); ysch929@nate.com (Y.S.C.); 2 Hanwha Systems Coporation, 244, 1 Gongdanro, Gumi, Gyeongsangbukdo 39376, Korea

**Keywords:** UAV, LWIR/MWIR, FLIR, TADS, NUC, adaptive filtering, image quality evaluation

## Abstract

Unmanned aerial vehicles (UAVs) are equipped with optical systems including an infrared (IR) camera such as electro-optical IR (EO/IR), target acquisition and designation sights (TADS), or forward looking IR (FLIR). However, images obtained from IR cameras are subject to noise such as dead pixels, lines, and fixed pattern noise. Nonuniformity correction (NUC) is a widely employed method to reduce noise in IR images, but it has limitations in removing noise that occurs during operation. Methods have been proposed to overcome the limitations of the NUC method, such as two-point correction (TPC) and scene-based NUC (SBNUC). However, these methods still suffer from unfixed pattern noise. In this paper, a background registration-based adaptive noise filtering (BRANF) method is proposed to overcome the limitations of conventional methods. The proposed BRANF method utilizes background registration processing and robust principle component analysis (RPCA). In addition, image quality verification methods are proposed that can measure the noise filtering performance quantitatively without ground truth images. Experiments were performed for performance verification with middle wave infrared (MWIR) and long wave infrared (LWIR) images obtained from practical military optical systems. As a result, it is found that the image quality improvement rate of BRANF is 30% higher than that of conventional NUC.

## 1. Introduction

**Figure 1:**
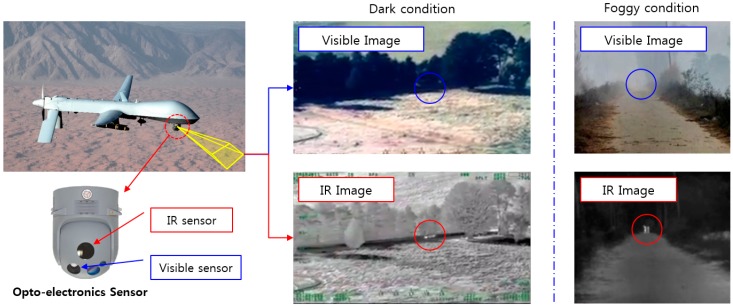
Advantages of using IR images in a general electro-optical sensor system [2].

Electro-optical infrared (EO/IR), target acquisition and designation sights (TADS), and forward looking infrared (FLIR) sensors are mounted on weapons systems such as unmanned aerial vehicles (UAVs), unmanned ground vehicles (UGVs), combat planes, and helicopters. Weapons systems acquire a tactical map of the battlefield or critical data for tracking and attacking targets using electro-optical sensors. Electro-optical sensor systems used in weapon systems are required to provide continuous high-quality images in order to detect the target even in low-visibility and foggy conditions. However, images from general daylight cameras cannot clearly show a target in dark or foggy conditions, as illustrated in Figure 1. Therefore, IR cameras are used in addition to daylight cameras, to improve the visibility for detecting and tracking targets in severe environments [1]. However, various types of noise are generated in IR images due to the complex semiconductor structures of IR sensors, which require high-level manufacturing processes. Additional noise is generated in IR images when such cameras are employed in severe environments, such as battlefields. Such noise significantly degrades the performance of target detection and tracking.

There are many methods to remove noise from images, and the methods applied to remote sensing images are similar to those applied to thermal images. Xinxin Liu et al. proposed methods to remove striped noise from remote sensing images [3], and Qiangqiang Yuan et al. proposed a method to remove dot- and line-type noise from hyperspectral images [4]. However, the noise in images obtained by practical military optical systems contains various additional types of noise, such as flickering noise, oblique line noise, long term variant (LTV) noise, and wavefront aberration. Therefore, noise filtering methods for military optical systems take various different approaches.

In images from military optical systems, general noise can be corrected using nonuniformity correction (NUC) and the neighbor pixels duplication (NPD) technique, but there remained unresolved noise problems [5,6,7]. The first type of noise consists of dynamic flickering dot and line noise, generated due to the semiconductor design characteristics of the focal plane area (FPA) and readout integrated circuit (ROIC) of IR sensors [8]. Noise of the second type is generated due to optical wavefront aberrations occurring because of the atmosphere and heat sources between the IR sensor and the target. The third type of noise consists of striped noise generated because of the use of the scan mirror method or power disturbances [9,10]. General noise that has been resolved and unresolved noise that requires solutions are summarized in Table 1.

**Figure 2:**
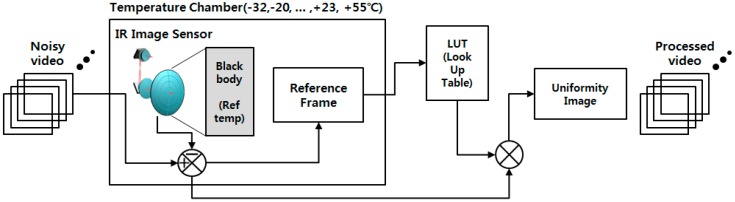
Block diagrams of the conventional NUC method.

In the case of thermal imaging, several studies have been conducted on compensating for nonuniformity characteristics. The NUC method is a representative method. A lookup table of noise is created using a black body that has constant temperature, and the noise is compensated using the lookup table [11]. The process of the NUC method is illustrated in Figure 2. However, the NUC method is incapable of removing noise such as moving dots and lines and wavefront aberration, listed in Table 1, because such noise can change rapidly. The LTV fixed pattern noise can be temporarily compensated by repeating NUC, but the noise compensation is not consistent. Also, the NUC method is not possible to implement in real time, because it requires an external black body. The two-point correction (TPC) method solves this problem by using a built-in thermal electric cooler (TEC) [12]. However, the TPC method requires complex hardware designs for optical structures. There have been studies regarding the compensation of noise without using a reference object, such as a scene-based NUC method and feedback-integrated scene cancellation (FiSC) [13,14]. Weighted nuclear minimization (WNNM) is a state-of-the-art method utilizing the low-rank-based image noise reduction method [15]. The advantages and disadvantages of these methods are summarized in Table 2.

**Figure 3:**
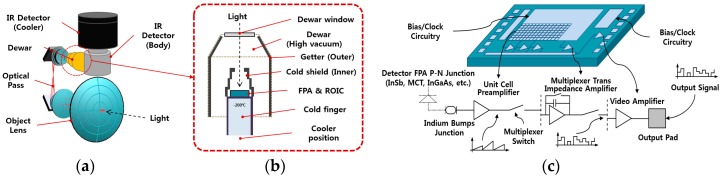
Design example of middle wave IR (MWIR) camera system: (**a**) Optical pass structures, (**b**) example of inner Dewar structures, (**c**) focal plane area (FPA) and readout integrated circuit (ROIC) schematic and electrical diagrams.

In this paper, the form and causes of three types of unresolved noise in IR images obtained by MWIR and LWIR sensors are analyzed. Thermal imaging camera systems are generally designed as shown in Figure 3a. The noise characteristics of output images depend on the properties of the semiconductor materials (InSb, MCT-HgCdTe, InGaAs, etc.), the characteristics of the peripheral circuits such as the ROIC and digital signal processing, and thermal detector elements such as the FPA. The light energy of an observed scene passes through the object lens. Then, it moves through the optical pass, and is projected onto the detector FPA. In addition, the temperature of a cooled MWIR/LWIR system drops to approximately −200 °C, improving the signal-to-noise ratio (SNR) to obtain high-resolution images. The representative types of cooler used in these systems are rotary (type-I: crank rotational driving refrigerant compression) and linear (type-II: piston reciprocating refrigerant compression). Noise can be generated by the motorized structures of cooler systems. The internal structure of the Dewar detector is illustrated in Figure 3b. The internal structure of the Dewar is maintained in a vacuum in order to prevent image quality degradation from scattering in air and incoming dust. There is a cold shield that prevents image disturbances resulting from unwanted heating, and the inner materials are covered with getter in order to minimize reflection. As shown in Figure 3c, the IR radiation projected onto the FPA is converted into the output signal on the ROIC. During this process, a dot-shaped noise occurs because of the lack of uniformity of the unit cell preamplifier, and vertical line-shaped noise occurs when the multiplexer impedance amplifier is not uniform.

In Section 2, a background registration-based adaptive noise filtering (BRANF) method is proposed to deal with these unresolved noises. BRANF performs background registration and principle component analysis (PCA) with continuous frames, which makes it possible to resolve the problematic noise listed in Table 1. Moving dots and lines and wavefront aberration are solved based on PCA, and LTV fixed pattern noise is compensated by converting it to short-term variant noise during the background registration process. In Section 3, quantitative image quality evaluation methods are proposed, which are capable of evaluating the improvement in images without using reference images. In Section 4, experiments using MWIR and LWIR images obtained from practical military optical systems are presented, and the performance is compared with the most commonly employed NUC method in practice and the state-of-the-art method WNNM.

## 2. Proposed Background Registration-Based Adaptive Noise Filtering Method

**Figure 4:**
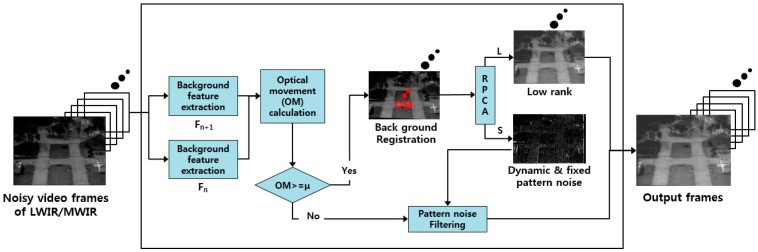
Block diagrams of proposed BRANF algorithm.

In this section, we describe the idea and method used to effectively remove noise, and explain the concept of an image-improvement technique using background registration-based adaptive noise filtering. The moving line- and dot-shaped noise types in the image are not fixed, and they rapidly change and move in a short time. The BRANF method is proposed, which can compensate for problematic noise such as moving dots and lines, wavefront aberrations, and LTV fixed pattern-type noise. The concept of an image improvement technique using BRANF is illustrated in Figure 4. First, noisy video frames ($F_{n}$) of LWIR/MWIR are given as the input of the image processing module. Feature points are extracted from two consecutive frame images using the Shi–Tomasi corner detector, which exhibits a good repeatability when the number of corners per frame is low [17]. Then, the optical flow is calculated based on the feature points, and the optical movement (OM) matrix is derived. It is important to calculate the correct OM in order to generate accurate background registration frame images [18]. Next, robust principle component analysis (RPCA) is performed [19,20,21,22,23,24]. This separates images into a low-rank part and a sparse part. The low-rank parts consist of images without dynamic pattern noise and fixed pattern noise, because fixed pattern noise is converted to dynamic patterns after background registration, whereas the sparse part contains all dynamic pattern noise and fixed pattern noise. If the OM value is larger than a certain threshold value (µ), a low-rank component becomes the output as the final corrected image, but the fixed pattern noise remains if the OM value is smaller than µ in the static line of sight (LOS). Therefore, a negative feedback process using the stored sparse component is designed to remove fixed pattern noise when the OM value is less than µ. The process of the algorithm is summarized in Algorithm 1. In Step 1, feature points of sequential frames ($F_{n}\;\mathrm{and}\;F_{n+1}$) are extracted. In Step 2, the background registration vector (*V*) is calculated using the Lucas–Kanade method [25]. Here, *i* is the number of pixels that a scanning window contains. If the size of the window is too small, then the feature extraction becomes poor, and if the size of the window is too big, the resolution of the optical flow decreases. Stabilized frame sequences are derived based on the background registration vector. In Step 3, the total image parts T are divided into a low-rank part and a sparse part. The number of images used for the PCA calculation should be carefully determined. If more images are used for the PCA calculation, the low-rank and sparse parts are well separated, but the computational complexity increases. Finally, in Step 4, if the OM is bigger than µ, then the low-rank part becomes the output image. If the OM is smaller than µ, then the sparse part is used to update the noise map, and noise filtering is performed on the input image. Then, the noise-filtered image becomes the output image.
**Algorithm 1. Background Registration-Based Adaptive Noise Filtering**Step 1.Background feature extraction between $F_{n}$ and $F_{n+1}$ where *F* denotes frames as shown in Figure 4.Step 2.Measurement of OM for background registration:
$$\mathrm{OM}=\left[\begin{array}{c} V_{x}\\ V_{y} \end{array}\right]={\left[\begin{array}{cc} \overset{i}{\underset{i=1}{\sum }}D_{x}{\left(m_{i}\right)}^{2} & \overset{i}{\underset{i=1}{\sum }}D_{x}(m_{i})D_{y}\left(m_{i}\right)\\ \overset{i}{\underset{i=1}{\sum }}D_{y}(m_{i})D_{x}\left(m_{i}\right) & \overset{i}{\underset{i=1}{\sum }}D_{y}{\left(m_{i}\right)}^{2} \end{array}\right]}^{-1}\left[\begin{array}{c} -\overset{i}{\underset{i=1}{\sum }}D_{x}(m_{i})D_{t}\left(m_{i}\right)\\ -\overset{i}{\underset{i=1}{\sum }}D_{y}(m_{i})D_{t}\left(m_{i}\right) \end{array}\right]$$
where $D_{x},D_{y},\;\mathrm{and}\;D_{t}$ are the partial derivatives of the images with respect to variable *x*, *y*, and *t*, respectively; *OM* is the optical movement vector; and *Vx* and *Vy* are local flow vectors. 
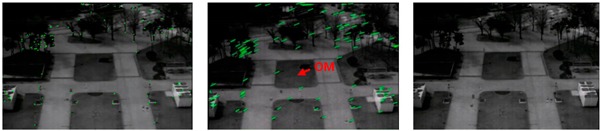

$$\begin{array}{ccccc} D_{x}(m_{1})V_{x} & + & D_{y}(m_{1})V_{y} & = & -D_{t}(m_{1})\\ D_{x}(m_{1})V_{x} & + & D_{y}(m_{1})V_{y} & = & -D_{t}(m_{1})\\ \vdots & & \vdots & & \vdots \\ D_{x}(m_{i})V_{x} & + & D_{y}(m_{i})V_{y} & = & -D_{t}(m_{i}) \end{array}$$
where the green colors are optical flows, the red arrow is the OM, and$m_{1},\;m_{2},\;\cdots ,\;m_{i}$ are the *i*th pixels on the window area.Step 3.Separate into sparse and low-rank parts using principle of robust PCA:
$$\min_{X,E}\;{\left||L|\right|}_{*}+\lambda {\left||S|\right|}_{1}\;\mathrm{subject}\;\mathrm{to}\;T=L+S$$
(L: low rank part, S: sparse part, λ: coefficient to control the amount of sparsity S).

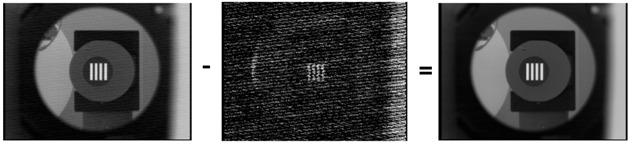
T: total imageS: sparse componentL: low-rank componentStep 4.If OM≥μ, output images can be low-rank images. Else, if OM < μ, output images are created by filtering noise using the updated noise map (S). (μ: threshold value in pixels.)

## 3. Quantitative Evaluation Method

An improved image can be determined by one’s sight. However, evaluating the improvement of the image is difficult. Therefore, a quantitative evaluation method for correct image quality evaluation is required. Signal-to-noise ratio (SNR) and peak signal-to-noise ratio (PSNR) are popular evaluation methods. However, these methods require a ground truth image as a reference in order to verify the extent to which the image is improved. Usually, there are no ground truth images available for practical images. Therefore, quantitative evaluation methods that can operate without a ground truth image are proposed in this paper.

### 3.1. SSNR Index

**Figure 5:**
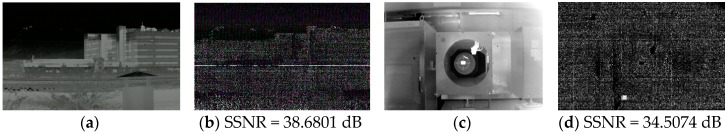
An example of evaluating the noise in MWIR images using subtraction signal-to-noise ratio (SSNR). Here, (**a**,**c**) are original images; (**b**) shows noise components containing horizontal line flickering noise; and (**d**) shows noise components containing horizontal/vertical line and dot flickering noise.

Subtraction signal-to-noise ratio (SSNR) is based on a subtraction method to find moving noise and noise patterns. The mean subtraction signal error (MSSE), which is an index to derive SSNR, is defined in Equation (1), and SSNR is defined in Equation (2).

(1) $$MSSE=\frac{1}{m\times n}\overset{m-1}{\underset{i=0}{\sum }}\overset{n-1}{\underset{j=0}{\sum }}{\left[K_{t}\left(i,j\right)-K_{t+1}\left(i,j\right)\right]}^{2}\;,$$

(2) $$\mathrm{SSNR}=10\log_{10}\left(MAX_{I}/MSSE\right),$$

where (*m*, *n*) is the image size, $K_{t}$ and $K_{t+1}$ are consecutive noisy images, and $MAX_{I}$ is the maximum pixel intensity of an image. An example of an image quality evaluation using SSNR is shown in Figure 5.

### 3.2. SNM Index

**Figure 6:**
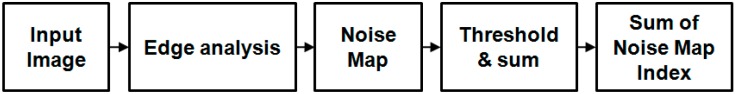
Measurement principle for a sum of noise map (SNM) index.

The quantity of image noise can be measured using a sum of noise map (SNM) index. A block diagram of this method is presented in Figure 6.

**Figure 7:**
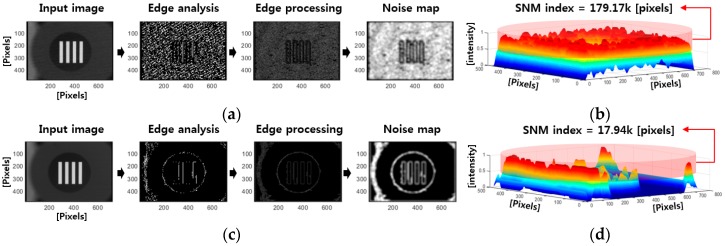
Measurement of noise map of an MWIR image using SNM: (**a**) noisy image; (**c**) noise-filtered image; (**b**,**d**) SNM measurement results.

By evaluating the quantity of edges using the aforementioned procedure, it is possible to confirm the distributions of noise in an image. Figure 7 presents the results for images containing simulated noise [16].

### 3.3. VSSIM Index

In general, structural similarity (SSIM) is used to evaluate the quality of an image based on a ground truth (GT) image. However, a GT image does not exist in thermal-imaging systems because the noise is not fixed. The proposed variant SSIM (VSSIM) focuses on the fact that humans recognize noise from blurring, flickering, wavefront aberration, and streaming patterns that are not within the background range. Therefore, the previous frame image is considered as the GT image, and the noise values are calculated as follows [26]:

(3) $$\mathrm{VSSIM}(x_{0},x_{1})=[l\left(x_{01,}x_{1}\right){]}^{\alpha }\;\cdot \;[c\left(x_{01,}x_{1}\right){]}^{\beta }\cdot {\left[s\left(x_{01,}x_{1}\right)\right]}^{\gamma },$$

where l, c, and s, are luminance, contrast, and structural terms, respectively,

(4) $$l(x_{0},x_{1})={\frac{2\mu_{x_{0}}\mu_{x_{1}}+C_{1}}{\mu_{x_{0}}^{2}+\mu_{x_{1}}^{2}+C}}_{1}$$

(5) $$c(x_{0},x_{1})=\frac{2\sigma_{x_{0}}\sigma_{x_{1}}+C_{2}}{\sigma_{x_{0}}^{2}+\sigma_{x_{1}}^{2}+C_{2}}$$

(6) $$s(x_{0},x_{1})=\frac{\sigma_{x_{0}x_{1}}+C_{3}}{\sigma_{x_{0}}\sigma_{x_{1}}+C_{3}}$$

(7) $$\mathrm{VSSIM}(x_{0},x_{1})=\frac{\left(2\mu_{x_{0}}\mu_{x_{1}}+C_{1}\right)\left(2\sigma_{x_{0}x_{1}}+C_{2}\right)}{\left(\mu_{x_{0}}^{2}+\mu_{x_{1}}^{2}+C_{1}\right)\left(\sigma_{x_{0}}^{2}+\sigma_{x_{1}}^{2}+C_{2}\right)}$$

**Figure 8:**
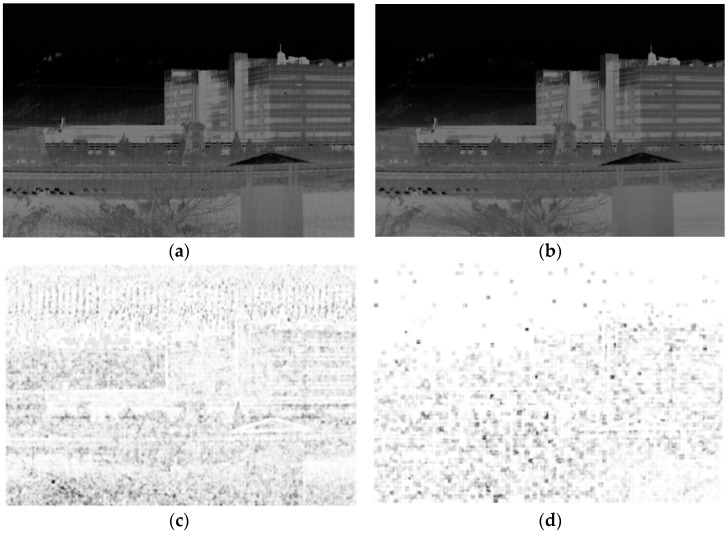
Noise-measurement variant structural similarity (VSSIM) index: (**a**) original noisy image; (**b**) denoised image; (**c**) noise map of the original noisy image (VSSIM = 0.8814); (**d**) noise map of denoised image (VSSIM = 0.9947).

Here, $\mu_{x_{0}},\mu_{x_{1}}$ are local means, $\sigma_{x_{0}},\sigma_{x_{1}}$ are standard deviations, and $\sigma_{x_{0}x_{1}}$ is the cross covariance for the images $x_{0}$ and $x_{1}$. In addition, $C_{1}={\left(0.01\times L\right)}^{2}$, where L is the specified dynamic range value; $C_{2}={\left(0.03\times L\right)}^{2}$, where L is the specified dynamic range value; and $C_{3}$ = $C_{2}$/2. Based on the changes in luminance, contrast, and structures of noise, VSSIM evaluates the image quality. VSSIM evaluation and the noise map are illustrated in Figure 8. Here, the denoised image is obtained by noise filtering using the proposed BRANF algorithm. The VSSIM index value is a decimal value between −1 and 1, and a value of 1 is only reached when the two images are identical.

## 4. Experimental Results and Analysis

**Figure 9:**
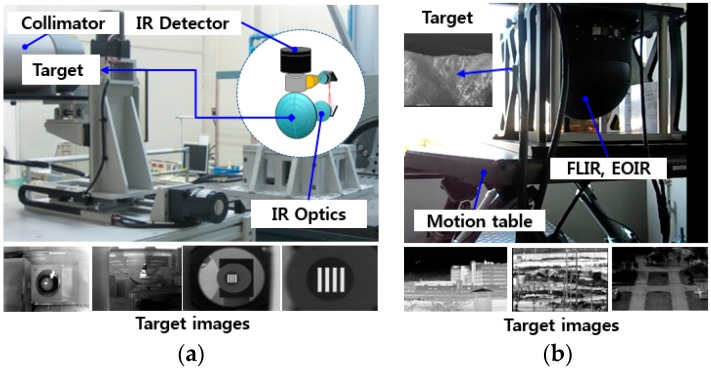
Experiment configurations: (**a**) An observation environment for simulation targets on a collimator with forward looking IR (FLIR) and electro-optical IR (EOIR); (**b**) an observation environment for actual targets.

We conducted an experiment to verify the noise reduction performance. NUC is the most popular method applied in practical military optical systems, and WNNM is the result of one of the latest studies on noise filtering. Therefore, these two methods are compared with the proposed BRANF method. The images used in the experiment are obtained from practical military optical systems using MWIR and LWIR containing various types of noise. The experimental configuration was installed as shown in Figure 9. Figure 9a shows the configuration for observing a four bar target of a collimator with an infinite optical distance. This is used to measure the minimum resolvable temperature difference (MRTD), in order to quantitatively determine the detection and recognition capabilities of the thermal camera. Figure 9b shows the configuration for observing outdoor scenes, such as buildings and mountains, without optical targets.

**Figure 10:**
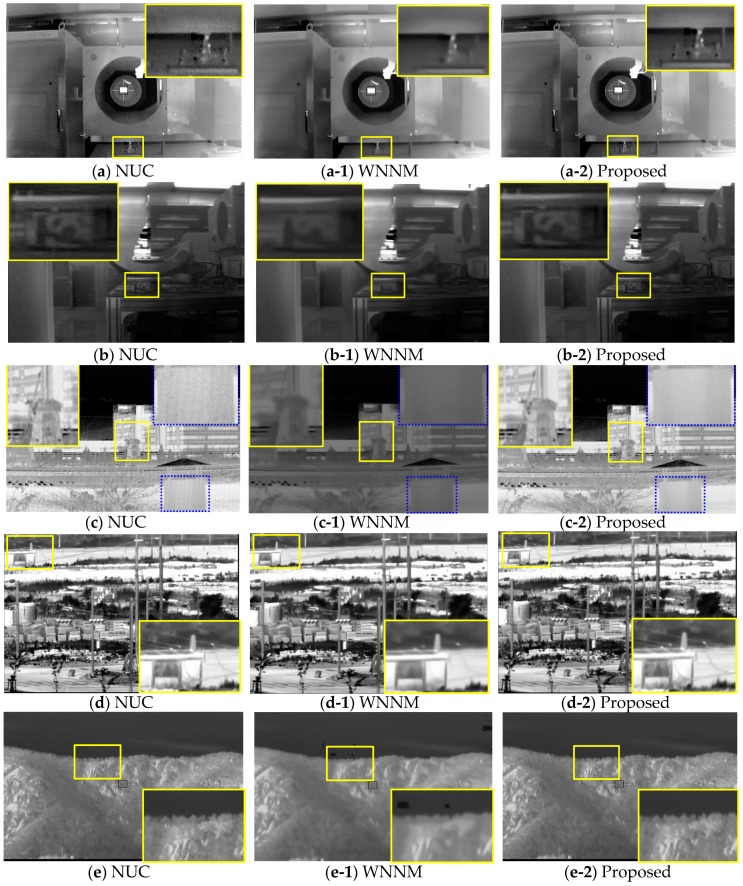

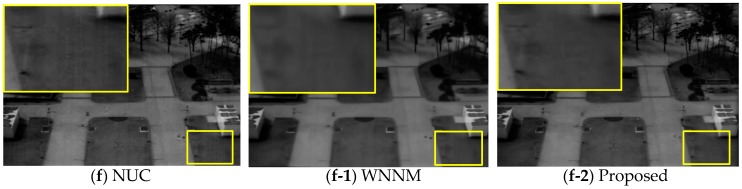
NUC, WNNM, and proposed the BRANF applied on MWIR and LWIR images with various types of noise: (**a**,**b**) flickering lines and dots; (**c**) vertical oblique lines; (**d**,**e**) wavefront aberration; (**f**) LTV line noise. (Experimental data set 1).

**Figure 11:**
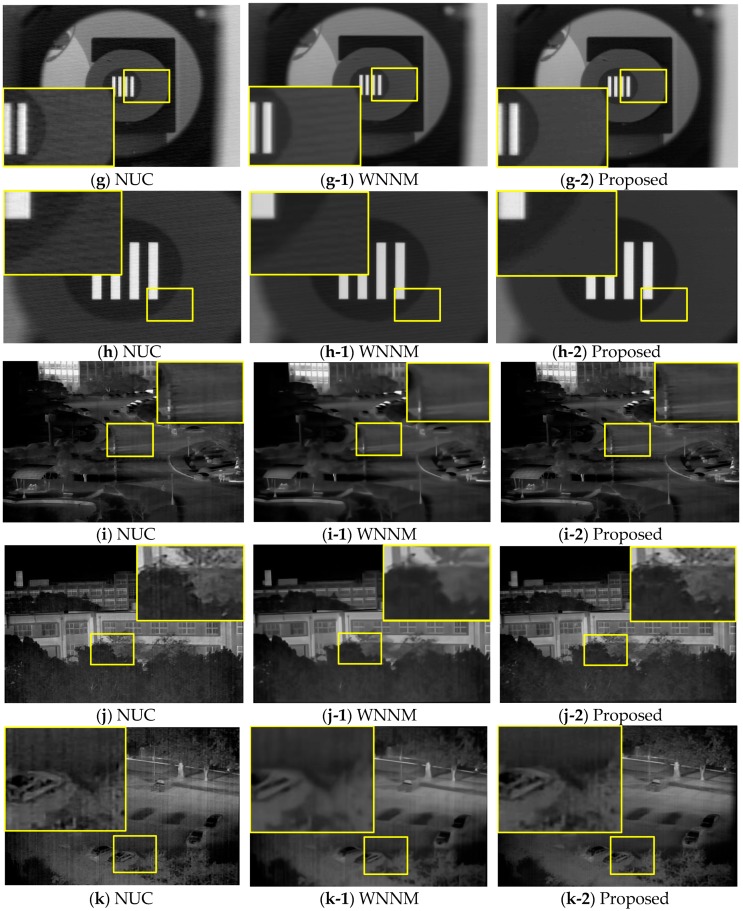

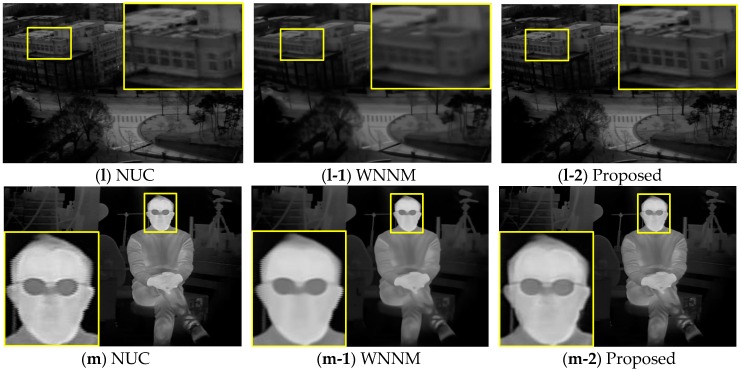
NUC, WNNM and proposed BRANF applied on MWIR, LWIR images with various types of noises: (**g**,**h**) moving oblique line noise; (**i**–**k**) LTV vertical line noise; (**l**) little noise case; (**m**) nonfixed jitter noise. (Experimental data set 2).

Figure 10 and Figure 11 show the results for NUC, WNNM, and the proposed BRANF on various types of noise. Figure 10a,b show the results for MWIR indoor images with flickering line and dot noise. Figure 10c shows an outdoor LWIR image with horizontal/vertical line noise occurring as a result of the mirror scanning method of the LWIR. Figure 10d,e are outdoor MWIR images with wavefront aberration, and Figure 10f is an LWIR outdoor image with LTV line noise. The subwindows in the figures are zoomed to show the detailed differences for each method. In general, BRANF removes more noise than NUC, and remains sharper than WNNM.

Figure 11g,h show the results for MWIR indoor images with oblique line noise occurring because of disturbed currents in the ROIC, the digital signal processor, or the power source. Figure 11i–k show LWIR images with vertical line noise. Figure 11l is an LWIR image with little noise, but demonstrates the sharpness of the resulting images. Figure 11m is an indoor LWIR image with jitter noise.

**Figure 12:**
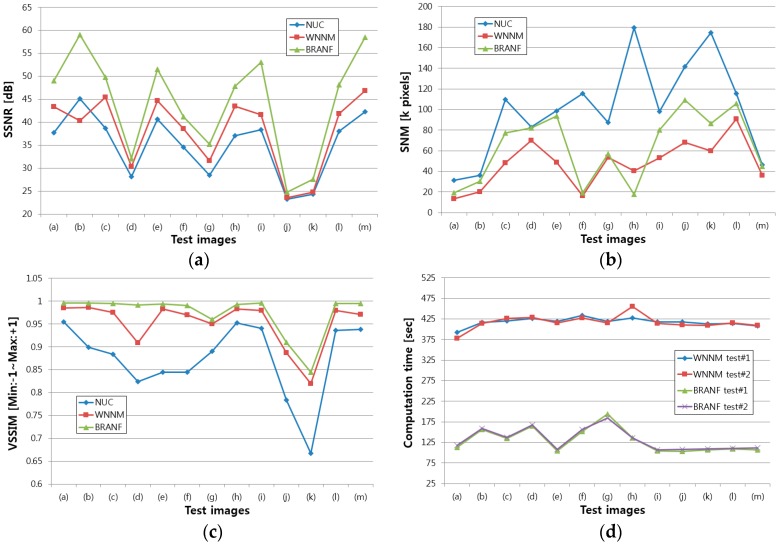
(**a**) SSNR value; (**b**) SNM value; (**c**) VSSIM value; (**d**) computation time of WNNM and BRANF.

**Figure 13:**
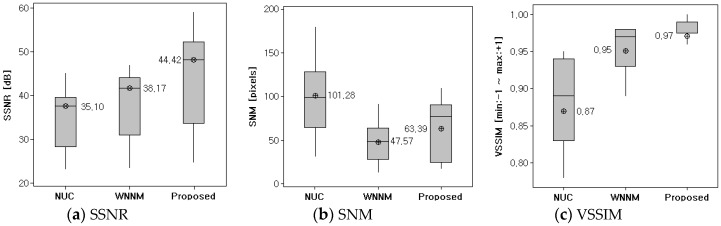
Boxplots and mean values of experimental results. (**a**) SSNR result; (**b**) SNM result; (**c**) VSSIM result.

Figure 12 shows the result of the three proposed quantitative evaluation methods applied to the images from Figure 10a to Figure 11m. The values of SSNR and VSSIM are higher when the noise is filtered more. The value of SNM is higher when there is more noise in the resultant image. In Figure 12a,c, the proposed BRANF shows the highest index value for SSNR and VSSIM. However, in the case of SNM in Figure 12b, BRANF is higher than WNNM, which appears to be the result of WNNM having less noise than BRANF. Here, the effect of the three indices should be considered. SSNR is effective in evaluating dynamic flickering noise. SNM is effective in evaluating fixed pattern noise, but has a problem that the result is good when the image is blurred. VSSIM is effective in evaluating structural noise, such as wavefront aberration. Therefore, the three evaluation methods are considered altogether to evaluate the performance of the noise reduction. As shown in Figure 10 and Figure 11, the resultant images for WNNM are more blurred than for BRANF. Therefore, there are less edges left in the resulting images, and so the SNM value is lower than that of BRANF. Even though the SNM value of BRANF is higher than that of WNNM, BRANF is more effective in applications such as target detection and tracking, because the resulting image remains sharp. For example, the result of SNM on Figure 11k shows the best performance with WNNM. The result of WNNM can be determined clearer by one’s sight. However, as shown in Figure 11k-1, it is hard to recognize the car because the image is blurred. The values of the indexes in Figure 12 are shown in Table 3 and the statistical analysis is shown in Figure 13.

## 5. Conclusions

In this paper, various types of noise and their causes in images from practical military optical systems have been analyzed. The advantages and disadvantages of conventional methods of removing such noise were also analyzed. BRANF was proposed to overcome the limits of conventional methods. Furthermore, the quantitative evaluation methods SSNR, SNM, and VSSIM were proposed, which can analyze the improvements of images quantitatively without ground truth images. The proposed BRANF method was compared with a widely employed method, NUC, and a recent method, WNNM, whose principle is similar to that of the proposed method. BRANF exhibited better values for SSNR and VSSIM, but the SNM was lower than that of WNNM. However, this is because the resulting images for WNNM were more blurred than those of BRANF. The resulting images for WNNM are more suitable for human viewing, but BRANF is better to use in applications such as detection and tracking. The computational load of BRANF was lower than that of WNNM. Real-time processing is left for future work. Dividing images into patches and applying parallel processing will improve the computational speed of BRANF. In addition, improving the background registration using other feature detection methods and background modeling can also improve the performance of BRANF. It can be expected that BRANF will improve the detection and recognition performance of defense systems using MWIR and LWIR.

## Figures and Tables

**Table 1:** Problematic noise types and methods for addressing them: Non-uniformity correction(NUC), two-point correction(TPC), scene based NUC(SBNUC), feedback-integrated scene cancellation(FiSC), weighted nuclear minimization(WNNM) and the proposed method.

Categories	Noise Types	Examples	Noise Filtering Methods (O: Perfect, △: Partial, X: Incomplete Solutions)					
			NUC [2]	TPC [4]	SBNUC [5]	FiSC [6]	WNNM [16]	Proposed
	Fixed pattern (grid, cloud, etc.)	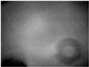	O	O	O	O	O	O
Basic Types of Noise	Shading phenomenon	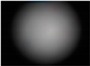	O	O	O	O	O	O
	Dead pixels	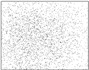	O	O	O	O	O	O
	① Moving dots and lines	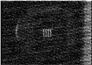	X	△	O	X	O	O
Problematic Noise	② Wavefront abberation	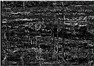	X	X	X	X	X	O
	③ Long term variant (LTV) fixed pattern noise	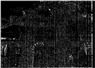	X	△	△	△	△	O

**Table 2:** IR image quality improvement methods: Non-uniformity correction(NUC), two-point correction (TPC), scene based NUC (SBNUC), feedback-integrated scene cancellation (FiSC), weighted nuclear minimization(WNNM) and the proposed background registration-based adaptive noise filtering (BRANF) method.

Methods	Summary Description	Advantage	Disadvantage
NUC [2]	One reference method using black body	Simple software design	Long process time with thermal chamber facilities Time/thermal variable noise
TPC [4]	Two reference method using built-in thermal electric coolers (TECs)	Short process time	Complex hardware design Periodic operation
SBNUC [5]	Background motion and sequence-based nonuniformity correction	Simple hardware design Real time compensation	Correction limits on stationary backgrounds Bluring, ghost, and fading problems
FiSC [6]	Uniformity correction based on time shift estimate of two frames	Short-term variable noise cancellation	Essential background motion Bluring and ghost problems
WNNM [16]	Low rank-based image noise reduction methods	Bad fixel (moving dots) replacement High peak signal-to-noise ration (PSNR) performance	Limits on mophological and time variant noise
Proposed BRANF	Dynamic, abberation, and long-term variable noise reduction using adaptive filtering algorithms	Both dynamic and static noise reduction. Minimize loss of original image	Computational complexity

**Table 3:** Quantitative measurement values comparing NUC and WNNM methods.

LWIR/ MWIR1 (Targets)	Images (Figure 10 and Figure 11)	Noise Types	SSNR (↑)			SNM (↓)			VSSIM (↑)		
			NUC [2]	WNNM [16]	Proposed	NUC	WNNM	Proposed	NUC	WNNM	Proposed
MWIR (Room)	(a)	Lines, dots	37.65	43.31	48.98	31.44	13.48	19.39	0.95	0.98	1.00
MWIR (Room)	(b)	Lines	45.10	40.30	59.00	36.30	20.00	30.54	0.89	0.98	0.99
LWIR (Field)	(c)	Oblique lines	38.68	45.44	49.75	109.63	48.05	77.34	0.88	0.97	0.99
MWIR (Field)	(d)	Wavefront aberration	28.12	30.29	32.08	82.82	69.91	82.14	0.82	0.91	0.99
MWIR (Field)	(e)	Wavefront aberration	40.62	44.66	51.49	98.79	48.65	93.85	0.84	0.98	0.99
LWIR (Field)	(f)	LTV lines	34.51	38.60	41.15	115.37	16.50	19.38	0.84	0.97	0.99
MWIR (Wide FOV2)	(g)	Oblique lines	28.40	31.56	35.20	87.49	53.54	56.83	0.89	0.95	0.96
MWIR (Narrow FOV)	(h)	Oblique lines	37.08	43.42	47.82	179.17	40.34	17.94	0.95	0.98	0.99
LWIR (Field)	(i)	LTV lines	38.36	41.64	53.04	97.90	52.89	80.24	0.94	0.98	0.99
LWIR (Field)	(j)	LTV lines	23.26	23.53	24.75	141.67	68.17	109.14	0.78	0.89	0.91
LWIR (Field)	(k)	Severe lines	24.31	24.73	27.53	174.48	59.83	86.58	0.66	0.82	0.84
LWIR (Field)	(l)	Less lines	38.05	41.86	48.13	115.50	91.03	105.91	0.93	0.98	0.99
LWIR (Room)	(m)	Jitters	42.27	46.86	58.51	46.06	36.08	44.77	0.94	0.97	0.99

^1^ Middle/long wavelength infrared (MWIR/LWIR), ^2^ field of view (FOV), 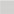: the best performance of each measurement.

## Supplementary Materials

Supplementary File 1
