# Supplementary figures and images for: Background Registration-Based Adaptive Noise Filtering of LWIR/MWIR Imaging Sensors for UAV Applications
Source: Sensors (Basel). 2017 Dec 27;18(1):60. doi: 10.3390/s18010060 (PMC5795610; doi:10.3390/s18010060)

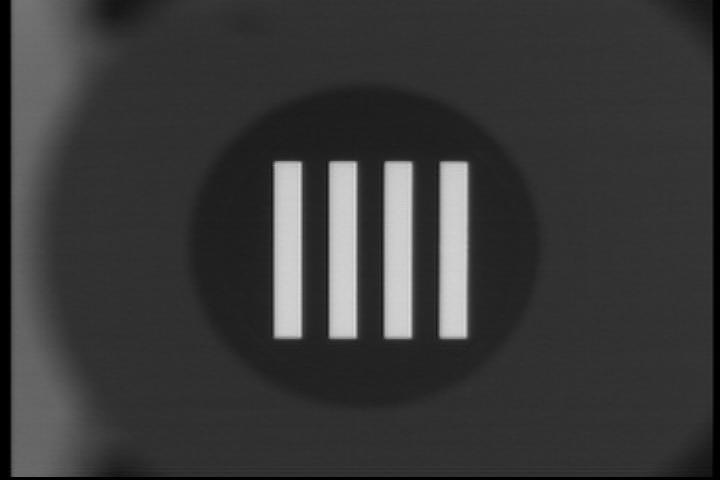

Supplement: Supplementary File 1 [file sensors-18-00060-s001.zip › Figure12(p).jpg]

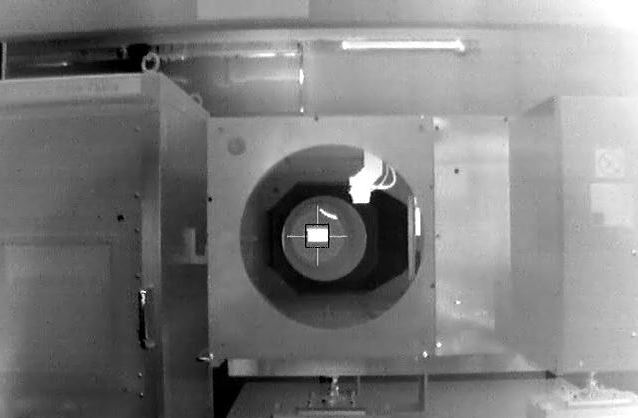

Supplement: Supplementary File 1 [file sensors-18-00060-s001.zip › Figure11(a).jpg]

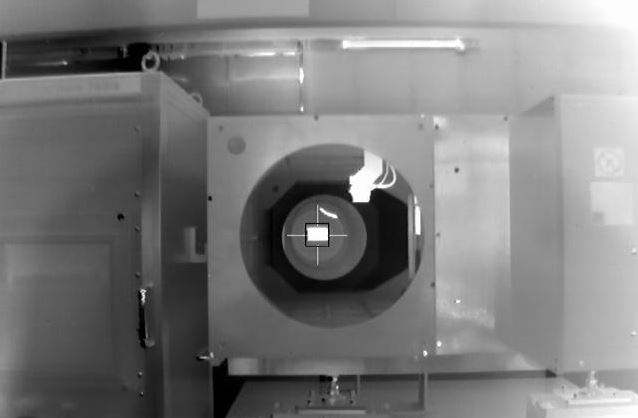

Supplement: Supplementary File 1 [file sensors-18-00060-s001.zip › Figure11(b).jpg]

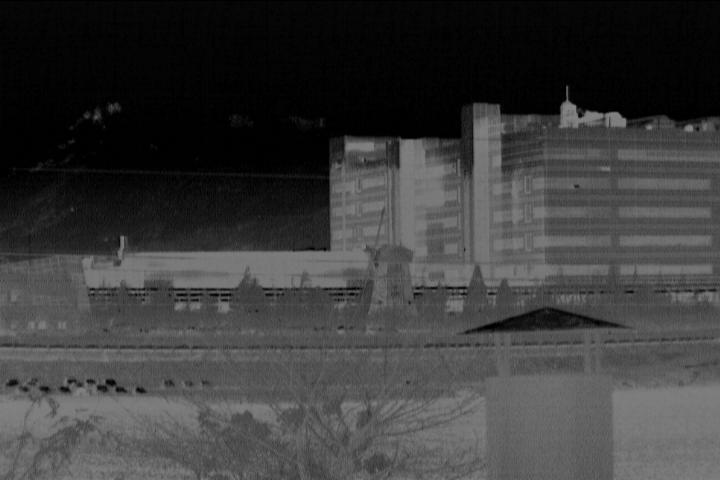

Supplement: Supplementary File 1 [file sensors-18-00060-s001.zip › Figure11(e).jpg]

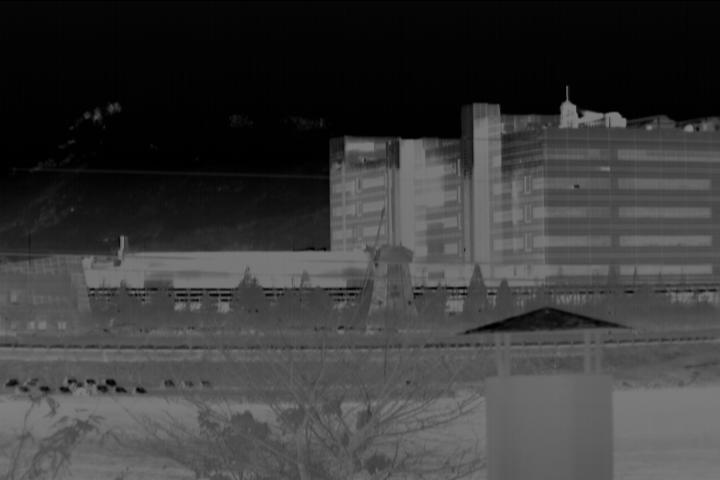

Supplement: Supplementary File 1 [file sensors-18-00060-s001.zip › Figure11(f).jpg]

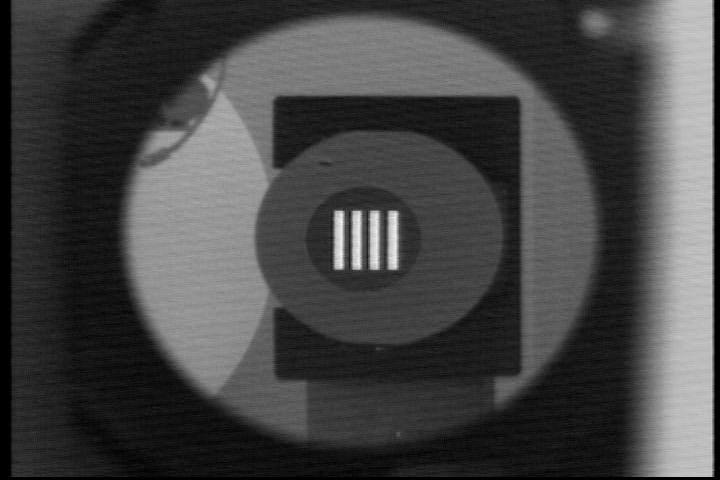

Supplement: Supplementary File 1 [file sensors-18-00060-s001.zip › Figure12(k).jpg]

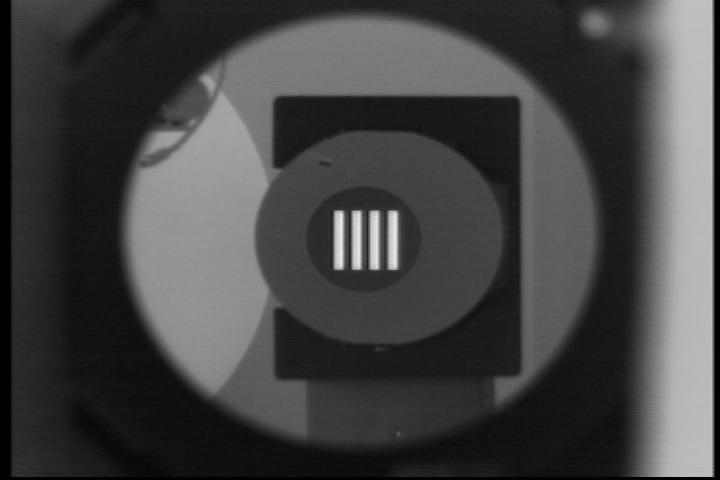

Supplement: Supplementary File 1 [file sensors-18-00060-s001.zip › Figure12(m).jpg]

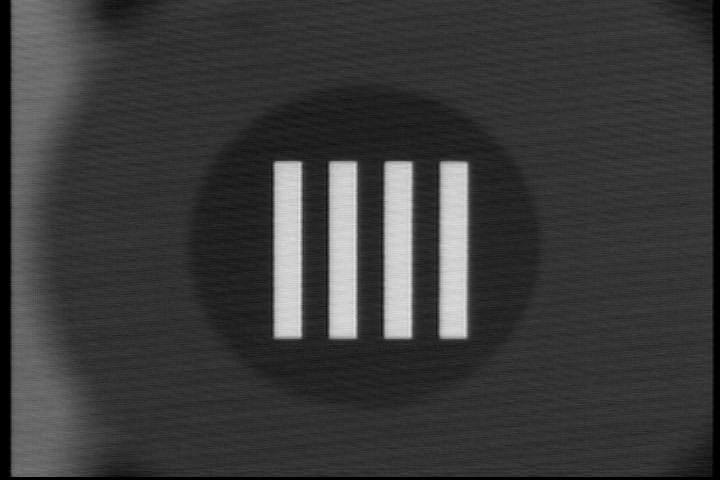

Supplement: Supplementary File 1 [file sensors-18-00060-s001.zip › Figure12(n).jpg]
